# A Curious Effect of Cocaine

**Published:** 1887-12

**Authors:** Frank Hamilton Potter

**Affiliations:** Lecturer on Laryngology, Medical Department, Niagara University; No. 42 West Tupper street


### *A CURIOUS EFFECT OF COCAINE.**

By FRANK HAMILTON POTTER, M. D.,

Lecturer on Laryngology, Medical Department, Niagara University.

The following case exhibits an effect of cocaine that is new to me, and, I believe, worthy of record:

M. R., aged twenty-four, female, married, presented herself suffering from follicular disease of the naso-pharynx, complicated by hypertrophies of the anterior ends of the turbinated bodies. The general health was good. Occasionally, there were slight disturbances of digestion, traceable to the disease just mentioned. The hypertrophies were removed with chromic acid, and the naso-pharyngeal disease treated with the glycerite of carbolized

* Reported to the Physicians' Club, September 23, 1887.

iodo-tannin, made according to Sajous' formula. This was changed at times for the stimulant astringents, ferric-alum being preferred. The patient was under observation for three months continuously, and, since, she has appeared now and then, as she deemed necessary. She is practically well; though, upon exposure to wet and cold, she is apt to observe some secretion from the naso-pharynx; and fearing a return of the disease, comes for treatment. Owing to these acute conditions, I have been able to prolong my observations, and to note well the following curious effect of cocaine: This drug was used in the strength of two and four per cent. at first, to overcome her fear of the instruments, and then in the various stages of the treatment, especially when using the chromic acid. Almost immediately upon each application—always within one or two minutes—she experienced a strong desire to have a movement of the bowels. Sometimes, she could not resist this desire; at other times, she was able to do so. After I became familiar with this result, I took great care to eliminate any source of error that might have entered into the observations. The desire always appeared upon the use of the drug, and varied in intensity with the amount used and the strength of the solutions. It would be slight and easily controlled, or strong and utterly beyond her control, according to the freedom with which the cocaine was applied. Other remedies, alone or in combination, applied in the same way with the same instruments, and with the intent to deceive, failed to produce the desire. When cocaine, however, was incorporated with these solutions, the phenomenon appeared just as if cocaine alone had been used.

In seeking for an explanation of this curious fact, we observe, first, that it is a nervous effect; the small quantity of the drug required, and the shortness of the interval from its application, preclude any other conclusion. The nerves supplying the nasal passages, aside from the nerve of special sense, are derived from the fifth nerve and the spheno-palatine ganglion of the sympathetic. We know that cocaine is a powerful stimulant to the vaso-motor nerves, and that it has an inhibitory action on the nerves of sensation. It cannot act, then, through the fifth nerve, as it destroys its power of carrying impressions. Therefore, we must conclude that it acts through the sympathetic system—that in this case, besides its stimulant action, it produces a distinct and peculiar impression, which is reflected to the nerve centers in the abdomen, and expends itself in the manner above described. Of course, it is useless to speculate as to what the peculiar impression may be. Until we can tell what nerve force itself is, we must despair of analyzing a special impression produced upon a special set of nerves, and acting in a special way. However, it would be interesting to know whether this action of cocaine is more universal than is supposed, and I trust any observations that may be made upon this subject will be reported.

No. 42 West Tupper street.

